# The Relationship Between Alcohol-Related Content on Social Media and Alcohol Outcomes in Young Adults: A Scoping Review

**DOI:** 10.35946/arcr.v45.1.04

**Published:** 2025-03-27

**Authors:** Mai-Ly N. Steers, Megan Strowger, Angela B. Tanygin, Rose Marie Ward, David A. Nolfi

**Affiliations:** 1 School of Nursing, Duquesne University, Pittsburgh, Pennsylvania; 2 Center for Alcohol and Addiction Studies, Brown University, Providence, Rhode Island; 3 Graduate College, University of Cincinnati, Cincinnati, Ohio; 4 Gumberg Library, Duquesne University, Pittsburgh, Pennsylvania

**Keywords:** alcohol, alcohol drinking, alcohol-related consequences, alcohol-related content, alcohol-related disorders, binge drinking, social media, young adult

## Abstract

**BACKGROUND:**

Alcohol-related content (ARC) is pervasive across social media. Existing research suggests that posting of and exposure to such content may affect young adults’ drinking and alcohol-related problems. However, a scoping review has yet to examine the literature within this field of research.

**OBJECTIVES:**

This scoping review delineates and describes the existing peer-reviewed quantitative research examining the associations between ARC posting and exposure and drinking and alcohol-related problems among young adults ages 18 to 30. Specifically, the authors sought to investigate (1) methodological trends in how exposure to and posting of ARC is assessed; (2) potential moderators of the association between exposure to and posting of ARC and drinking outcomes; (3) how exposure to and posting of ARC is associated with alcohol consumption and alcohol-related problems; and (4) potential gaps in the literature.

**ELIGIBILITY CRITERIA:**

This review includes original, empirical, quantitative studies, published in English from 2006 to 2023, that measured alcohol consumption and/or alcohol-related problems and the use of ARC on social media in 18- to 30-year-olds.

**SOURCES OF EVIDENCE:**

The authors systematically searched the PubMed, Cumulative Index to Nursing and Allied Health Literature (CINAHL), PsycInfo, and Scopus databases on May 30, 2023, and reran the searches on November 1, 2023.

**CHARTING METHODS:**

The authors designed a form to extract data and statistics related to alcohol drinking and ARC measures. Pairs of authors extracted the data for each study independently, and then a third author reviewed their work to resolve differences.

**RESULTS:**

In total, 3,112 papers were selected via preliminary search terms. After removing duplicates and other articles deemed ineligible based on screening articles at the title and abstract level as well as assessing full-text articles for eligibility (*n* = 3,079), the final review included 33 studies. Overall, the results of the scoping review revealed a lack of consistent definitions and standardized assessments related to ARC. Despite these factors, the authors uncovered robust positive relationships between posting ARCand drinking and alcohol-related problems. The literature also mostly found positive, significant linkages between exposure to ARC and drinking and alcohol-related problems.

**CONCLUSIONS:**

This scoping review highlights the need for consistentoperationalization and empirically validated measures related to ARC. In addition, the authors propose a theoretical model that may serve as a road map for future interventions targeting young adults.

## Rationale

Alcohol misuse among young adults is a major public health concern. Nearly 55% of adults ages 18 to 29 report consuming alcohol within the past month, and 31% report binge drinking (defined as four or more drinks for a woman and five or more drinks for a man within a 2-hour timeframe).^1^ However, trends differ within this age range, as the prevalence for past-month alcohol use is 64% for those ages 18 to 20 and 77% among 21- to 29-year-olds.^2^ Prevalence for any binge drinking is higher among young adults ages 18 to 21 (40.8%) than those ages 22 to 30 (28.3%).^3^ Additionally, 15% of young adults ages 18 to 29 have met criteria for alcohol use disorder,^4^ with prevalence at 19% among those ages 18 to 24 and 13% among those ages 25 to 29.^2^ Heavy drinking (e.g., binge drinking or consuming eight or more drinks per week for women and 15 or more drinks per week for men^5^) may lead to a multitude of short-term consequences (e.g., risky sexual behaviors,^6^ violence,^7^ hospitalizations^8^) and long-term outcomes (e.g., cancer,^9^ dementia,^9^ liver failure^9^), including death.^10^

According to a Pew Research Center poll conducted in 2023, 74% of adults under age 30 reported using at least five of the following social media platforms: Instagram, Snapchat, TikTok, Reddit, Twitter (X), YouTube, Pinterest, LinkedIn, WhatsApp, BeReal, and Facebook.^11^ Moreover, a study conducted by Scott et al. found that 55% of young adults ages 18 to 29 reported checking social media several times per day and spending 61 to 70 minutes on social media daily.^12^ Further, social media use has become the dominant method of communication among young adults since in-person social interactions began to decrease in this age group in the 2010s compared to generations of young adults in previous decades (1980s, 1970s);^13^ thus, social media is an integral form of communication for this demographic.

Alcohol-related content (ARC) is ubiquitous on young people’s social media feeds and has been linked to increases in consumption among young adults.^14^ ARC is defined as social media posts that feature, contain, or imply alcohol use. This includes, but is not limited to, posts by individual users, peers, news organizations, restaurants and bars, community groups, alcohol companies/brands, and social media influencers. Exposure to ARC involves viewing ARC posts circulated by the sources listed above. Conversely, posting ARC refers to generating one’s own content or sharing ARC from other sources.

Qualitative research in this domain has highlighted themes of social bonding, connectedness, and the normalization of drinking behaviors through sharing ARC on social media.^15^,^16^ Additionally, several theories have been proposed as possible explanations for the associations between ARC and young people’s drinking habits. Social learning theory posits that people learn through the process of observing, imitating, and modeling others’ behavior that they perceive to be socially desirable.^17^ Consequently, young people who view their peers’ ARC posts may be socially influenced to post similar content. In fact, according to a qualitative study that examined Facebook, Instagram, Snapchat, and Twitter, college students may construct an online identity by posting ARC that reflects a desirable social status and maintains relevance within a specific network.^16^ Relatedly, a content analysis of young people’s Facebook and Instagram profiles found that 97% of ARC portrayed drinking in a positive, social context (e.g., joyful celebrations involving alcohol).^18^ Another content analysis of college students’ Facebook posts revealed that images displaying alcohol in a positive light tended to garner more social validation from peers (i.e., likes).^19^

Recent evidence has indicated that young adults’ alcohol use is predictive of not only their future drinking, but also their prospective posting of ARC.^18^ Social norms theory proposes that young people may derive their drinking norms from ARC on social media.^20^,^21^ The more ARC they see, the more alcohol they may perceive their peers to consume, leading to an overinflation of peer drinking norms.^22^,^23^ Perceived peer drinking norms are a consistent predictor of young people’s alcohol consumption.^24^–^26^

According to the reinforcing spirals model, the more often young people who drink heavily post and are exposed to ARC, the more likely they are to develop an online and offline “drinker identity” (i.e., tendency to view themselves as a drinker) because they often receive social validation for doing so (i.e., engagement with their posts in the form of likes and comments).^27^ Others within the individual’s social media network who drink may see this validation and aspire to receive the same level of approval from their peers. Thus, they may initiate posting ARC of their own, which may lead to the adoption and bolstering of online and offline drinking identities among additional network members. As a result, ARC may lead to increases in alcohol use within young people’s social media networks by encouraging others to adopt and maintain these ARC-posting and heavy drinking behaviors. A 21-day diary study found that college students’ exposure to ARCposts on Facebook was associated with a greater probability of drinking on the same day.^28^ Thus, this online “culture of intoxication”^29^ may instigate, maintain, and sustain heavy drinking within a given network.

To date, several reviews, including meta-analyses,^14^,^30^ a systematic review,^31^ and nonsystematic reviews,^32^–^36^ have examined associations between ARC and alcohol use and alcohol-related problems among young adults. The meta-analyses and systematic review have synthesized the data involving the specific research question of whether ARC is linked to alcohol use and alcohol-related problems and found a consistent association between posting, exposure, and engagement with ARC and drinking.^14^,^30^,^31^ However, unlike the current review, most of these prior reviews (1) have not focused on methodological trends, aside from noting inconsistent ways of measuring ARC and drinking; (2) have not identified potential moderators of the association between ARC and drinking, which may make people more vulnerable to being socially influenced by ARC; (3) have approached the literature with a specific research question in mind rather than broadly examining and identifying potential gaps; and (4) have not captured the full age range of young adults (ages 18 to 30)^37^ or have also included adolescents, who are in distinctly different developmental periods compared to young adults. Within the alcohol literature, a large number of articles, including scoping reviews and trajectories over time, have pinpointed the 18 to 30 age range as being a critical time for examining developmental patterns.^38^–^41^ Arnett contends that the age range of 18 to 29 comprises a distinct transitional period characterized by identity exploration, instability, self-focus, feeling in-between, and possibilities.^42^ Taking both Arnett’s definition and the alcohol literature into account, the authors of this scoping review therefore examined the 18 to 30 age range to broadly encompass the period of young adulthood. Further, given that the social media landscape is ever evolving, the review encompassed both older and more recent articles.

## Objectives

To address existing gaps, this scoping review of peer-reviewed literature aimed to answer the question: What is the association between exposure to and posting of ARC and alcohol consumption and alcohol-related problems among young adults? Its goal is to inform both researchers and the broader public health community of the importance of assessing young people’s involvement with ARC on social media. Specifically, the authors sought to examine (1) methodological trends in how exposure to and posting of ARC is assessed; (2) potential mediators and moderators of the association between exposure to and posting of ARC andalcohol outcomes; (3) how exposure to and posting of ARC is associated with alcohol consumption and alcohol-related problems; and (4) potential gaps in the literature that would benefit from further research in the future.

## Methods

### Protocol and Registration

The authors wrote the protocol using guidance from the Preferred Reporting Items for Systematic Reviews and Meta-Analyses Extension for Scoping Reviews (PRISMA-ScR)^43^ and the Joanna Briggs Institute (JBI) Manual for Evidence Synthesis.^44^ The protocol was published on November 16, 2023, on the Duquesne Scholarship Collection (https://dsc.duq.edu/faculty/1373/).

### Eligibility Criteria

To be included in the review, studies needed to be original, empirical quantitative research that measured alcohol consumption and/or problems resulting from study participants’ use of alcohol-related social media content. Papers were included if they were published in peer-reviewed journals in English from 2006 to 2023 and included participants ages 18 to 30. Exclusion criteria included the following study designs: qualitative research, content analyses, systematic reviews, and meta-analyses. Although qualitative research offers valuable insights into the lived experience of particular groups of people, the findings may not be generalizable to the broader population, making it difficult to draw conclusions.

### Information Sources

To identify potentially relevant studies, the authors searched the following databases: the National Library of Medicine’s PubMed, which indexes the biomedical and life sciences literature; EBSCO Information Services’ Cumulative Index to Nursing and Allied Health Literature (CINAHL), which indexes literature related to nursing and allied health professions; the EBSCOhost version of the American Psychological Association’s PsycInfo, which indexes the psychological literature; and Elsevier’s Scopus, which indexes literature from virtually all major fields of study, including health sciences, social sciences, natural sciences, and humanities.

### Search

The entire research team discussed potential search terms, and the librarian member of the team drafted initial search strategies. After consulting with the whole team, the librarian revised each database’s search strategy. Finally, another librarian reviewed the search strategies for completeness.

The customized search strategies included keywords and phrases as well as database-specific subject headings related to ARC on social media as well as to alcohol consumption and alcohol-related problems (see Table 1 for search strategy by database).

The strategies included Boolean operators to combine terms and truncation to search for variant word endings with the goal of maximizing search recall.

The librarian conducted the searches on May 30, 2023, updated them on November 1, 2023, and subsequently uploaded them into the Covidence systematic review management system (https://www.covidence.org). Covidence then removed the duplicate citations.

After initially selecting studies for inclusion in the present review, the authors used Scopus to examine studies citing the selected research papers (known as “progeny”) and uploaded them into Covidence on November 30, 2023. The research team also reviewed references cited in the selected studies (known as “ancestry”) and uploaded them into Covidence as well. Searching progeny and ancestry references served as a final step to attempt to identify all relevant studies.

### Selection of Sources of Evidence

The authors used Covidence to facilitate the study selection process. To reduce bias, Covidence allows each reviewer to make a decision independently, without revealing their decisions to the rest of the team. Four authors screened potential studies’ titles and abstracts and reviewed full texts for inclusion or exclusion. The authors were grouped into two pairs. Half of the articles were reviewed by one pair, and the other half were reviewed by the other pair. At each step of the process, the two authors in each pair needed to agree to include or exclude each study. If they did not, Covidence added the study to a conflict resolution file. Conflicts were then resolved by a fifth author who was not part of the review process. After the initial full-text review, the authors further discussed reasons for excluding full-text articles and refined their process for assigning reasons for excluding full texts.

### Data Charting Process and Data Items

Four of the authors used Covidence to develop a data charting form that enabled them to review the full text of studies online, select data elements, and directly input them into a draft results table. The authors reviewed the characteristics of the studies, including country in which the study was conducted, research question/hypotheses, study design, number of participants, study inclusion and exclusion criteria, recruitment methods, population characteristics, ARC platforms studied, ARC measures, alcohol measures, and study outcomes.

Pairs of authors extracted the data for each study independently, and then a third author reviewed each pair’s extraction drafts and resolved any differences. After completing initial data extraction, two authors proposed adding sample statistics indicating heavy drinking, drinking inclusion criteria, and social media criteria to the charting. They further proposed adding chart elements for outcomes that included measures of ARC, which encompassed posting ARC (participant’s ARC posting) and exposure to ARC (viewing ARC posts from other sources). Additionally, the authors noted if engagement with ARC (defined as “interacting with” ARC, such as liking or commenting on posts) was also assessed within the studies. Other proposed chart elements included theoretical frameworks, types of analysis, and mediation/moderation models. The rest of the authors agreed and then revised the charting process to include these additional characteristics. A full list of chart elements, including study characteristics, methods and dates; participant characteristics; ARC and alcohol measures; and outcomes, is available from the corresponding author upon request.

### Synthesis of Results

Appendix 1 summarizes the characteristics of the included studies, including country, sample size (including whether it was a college student sample and/or a heavy-drinking sample), study design, sample demographics, ARC predictors, and alcohol outcomes. The column describing the outcomes also highlights whether positive, negative, or no association was found between the predictors and outcomes. Summaries of the study findings are described narratively in the “Results” section below.

## Results

### Selection of Sources of Evidence

**Figure 1:**
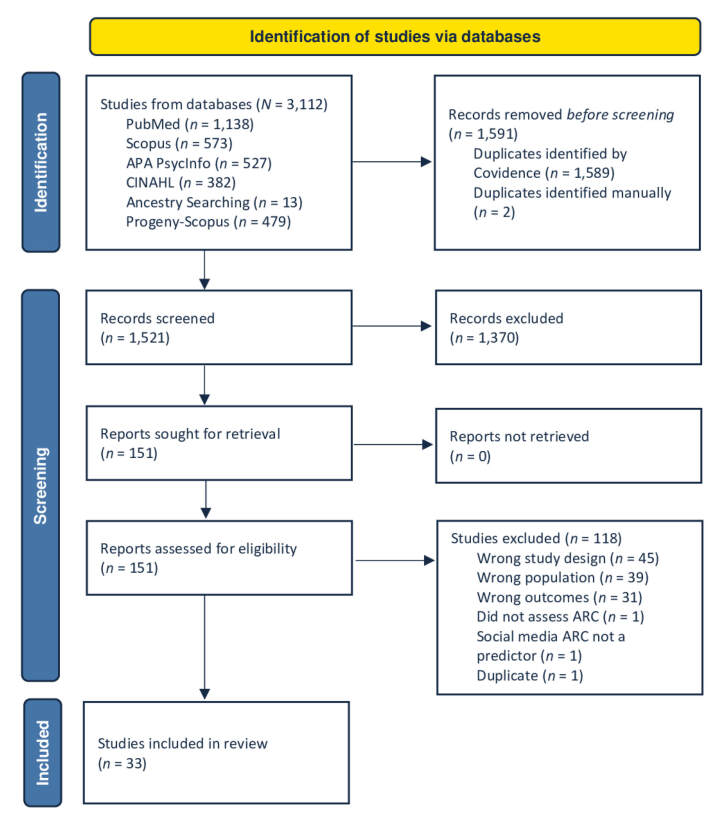
PRISMA 2020 flow diagram depicting the literature search and selection of articles. *Note:* ARC, alcohol-related content. *Source:* PRISMA flow diagram templates are distributed in accordance with the terms of the Creative Commons Attribution (CC BY 4.0) license. Page MJ, McKenzie JE, Bossuyt PM, et al. The PRISMA 2020 statement: An updated guideline for reporting systematic reviews. *Syst Rev*. 2021;10(1):89. https://doi.org/10.1186/s13643-021-01626-4.

The literature searches uncovered 3,112 articles. Covidence removed 1,589 duplicates, and the authors manually identified two additional duplicates. The remaining 1,521 articles were screened at the title and abstract level. Of these, 1,370 did not meet the inclusion criteria (e.g., regarding age, study design, or ARC exposure). Thus, 151 articles were retrieved and assessed for eligibility, of which 117 articles were excluded for the following reasons: 37 did not meet the age criteria, 33 did not address ARC or alcohol consumption and related problems, 45 were not quantitative studies, and one addressed the wrong population. The final review included 33 studies as illustrated in the PRISMA Flow Diagram^45^ (Figure 1).

### Theoretical Frameworks

Almost half of the studies^46^–^58^ did not specify any underlying theoretical framework for the association between ARC and alcohol outcomes; the others employed a wide range of theoretical frameworks. The most common theory used was social learning/cognitive theory^17^ (18% of studies), which suggests that modeling observed behavior in peers (i.e., drinking) has facilitated positive outcomes (i.e., friendship).^59^–^64^ Social norms theory^65^ was used in 15% of studies; it proposes that perceptions of how much others engage in a behavior (e.g., drinking/ARC) are associated with engagement in that behavior at a similar level.^61^,^66^–^69^ Alcohol expectancy theory/expectancy-value model,^70^ which suggests that modeling observed behavior in peers who do not experience negative consequences from the behavior (e.g., drinking/ARC) is associated with greater positive expectancies, was used in 9% of studies.^59^,^60^,^63^ Similarly, 9% of studies employed the Facebook influence model,^71^ which states that social media (e.g., ARC) influence offline behavior (e.g., drinking) through multiple factors such as users connecting with others.^58^,^72^,^73^ The prototype-willingness model^74^—which states that alcohol-related normative perceptions, personal attitudes, and prototypes are associated with willingness and intentions to drink/post ARC, which in turn predicts drinking behavior—was used in 6% of studies,^75^,^76^ as was the theory of planned behavior,^77^ which posits that alcohol-related norms and attitudes predict intentions, and these in turn predict drinking.^75^,^78^ Several frameworks were mentioned in one study each (3%), including social identity theory,^79^ which suggests that identities are derived from perceived group membership, and members modify their attitudes and behavior (e.g., drinking/ARC) to match the group;^80^ identity shift theory,^81^ which states that posting ARC online is more closely linked to a person’s own drinking behavior than to general social media posting behavior;^66^ uses and gratifications theory,^82^ which states that individuals who drink may actively seek out and engage with ARC online;^62^ theory of reasoned action,^83^ which suggests that exposure to ARC, friend approval for drinking, and personal attitudes toward alcohol predict drinking intentions, which in turn predicts drinking behavior;^84^ and social impact theory,^85^ which suggests that group membership can influence personal thoughts, behaviors, and attitudes related to drinking and ARC.^86^ Seven studies (21%) used multiple theories.^59^–^63^,^66^,^75^

### Characteristics of Participants

Perhaps because of the fact that the scoping review only included articles written in English, nearly three-fourths of the studies (73%) were conducted within the United States.^46^,^48^,^50^–^54^,^57^–^64^,^68^,^69^,^72^,^80^,^84^,^86^–^89^ The remaining studies were conducted in Belgium,^66^,^67^,^78^ the United Kingdom,^55^ Norway,^76^ Australia,^75^ Mexico,^47^ Kenya,^56^ and Uganda.^73^ The vast majority of the samples consisted of college student populations (82%).^47^,^48^,^50^–^54^,^56^–^61^,^63^,^64^,^66^,^68^,^72^,^73^,^75^,^76^,^80^,^84^,^86^–^91^Studies that did not solely utilize college student samples (18%)^46^,^51^,^55^,^62^,^69^,^78^ still reported the majority of their samples as being enrolled in college/university with the exception of one study.^69^ These six studies recruited their samples through paid online services,^46^,^69^ social media promotion,^55^,^78^ or both,^51^ or utilized a public school population initially recruited via email and phone for a larger longitudinal study.^62^

The bulk of the studies (88%) had a majority female-identified population.^46^–^48^,^50^–^55^,^57^–^64^,^66^,^68^,^69^,^72^,^75^,^76^,^78^,^80^,^84^,^86^–^89^ Of these, 10 studies (27%)^50^,^52^,^55^,^57^,^61^,^75^,^78^,^80^,^86^,^89^ included over 65% female-identified participants, and one study^75^ consisted solely of female-identified respondents. None of the studies reported participants’ sexual orientations. For all studies that reported participants’ race (79%),^46^,^48^,^50^–^55^,^57^–^64^,^68^,^69^,^72^,^75^,^86^–^89^ the majority of participants were White individuals (i.e., more than 45% White) with 11 studies (36%)^46^,^48^,^50^,^52^,^54^,^55^,^57^,^64^,^68^,^69^,^87^ including over 65% White participants. (Some studies used the term “Caucasian” within their racial/ethnic identification schema as a synonym for “White.”)

Upon examining sample statistics involving drinking, about one-third of the studies (30%) were classified as having a majority heavy-drinking sample.^48^,^50^,^55^,^57^,^60^,^69^,^75^,^76^,^78^,^86^ Based on the Centers for Disease Control and Prevention’s definition of heavy episodic drinking (i.e., four or more drinks per occasion for women, five or more drinks per occasion for men),^92^ the authors categorized six studies (18%)^48^,^50^,^57^,^60^,^69^,^86^ as having heavy-drinking samples because they reported mean values of five or more for the highest number of drinks consumed on a single day (peak drinks) or for drinks per occasion, heavy episodic drinking, or binge drinking.^93^ One of the six studies^86^ separately reported the means for peak drinks for individuals who did or did not post ARC; only people who posted ARC were found to engage in heavy alcohol use. Another study^76^ was classified as having a majority heavy-drinking sample because the researchers reported a mean total score of 8 on the Alcohol Use Disorders Identification Test (AUDIT), which the World Health Organization considers to be indicative of hazardous or harmful alcohol use and potentially indicative of alcohol dependence.^94^ Moreover, three studies^55^,^75^,^78^ were categorized as having heavy-drinking samples based on mean AUDIT-Consumption Subscale (AUDIT-C) scores that indicate increased risk for hazardous drinking or alcohol use disorder.^95^ One of these studies^75^ with a 100% female population was categorized based on the female AUDIT-C threshold score of 3 or more; the other two studies^55^,^78^ were categorized based on an AUDIT-C score of 4 or more, which encompasses the heavy drinking thresholds for both men and women.

The remaining studies either did not show evidence of a majority heavy-drinking sample (55%)^46^,^47^,^53^,^54^,^58^,^59^,^61^–^63^,^66^–^68^,^72^,^80^,^84^,^87^–^89^ or did not provide enough information to determine whether they had a majority heavy drinking sample (15%).^51^,^52^,^56^,^64^,^73^

### Participant Inclusion Criteria

In terms of qualifying participant measures, surprisingly, only four studies (12%) included some form of drinking criteria.^48^,^60^,^61^,^86^ Moreover, drinking criteria varied considerably among these studies. Only one study^60^ recruited students who drank heavily (i.e., those who engaged in heavy episodic drinking, defined as four or more drinks for women and five or more drinks for men at least once in the past month). Two studies^48^,^86^ required participants to have consumed any alcohol in the past month, and one study^61^ required students to have consumed alcohol within the past week; however, these studies did not require a specific quantity of drinks consumed.

With regards to social media, a little over one-third of studies (39%) explicitly specified inclusion criteria that required participants to possess and/or be an active user of a specific social media platform (24%)^48^,^54^,^58^,^63^,^66^,^80^,^87^,^88^ or of social media more generally (15%).^57^,^67^,^72^,^86^,^89^ The remaining studies (61%) did not unequivocally state that social media usage was a requirement for participation.^46^,^47^,^50^,^51^–^53^,^55^,^56^,^59^–^62^,^64^,^68^,^69^,^73^,^75^,^76^,^78^,^84^

### Study Designs

Twenty-three studies (70%) employed a cross-sectional study design.^46^–^48^,^51^–^53^,^55^–^57^,^61^,^62^,^64^,^66^,^68^,^69^,^73^,^75^,^76^,^78^,^80^,^86^,^89^ Nearly one-fourth of the studies (24%) utilized a longitudinal assessment.^50^,^58^–^60^,^63^,^72^,^84^,^88^ Three of the studies with longitudinal assessment^50^,^58^,^84^ also included assessment of participants’ ARC posts that were manually coded by researchers, and one study60 included a daily diary component. Five studies (15%)^51^,^62^,^66^,^80^,^87^ supplemented the cross-sectional component with manual coding of ARC, including one study^62^ that also implemented a daily diary study design. One study^67^ solely used a daily diary study design, and a single study performed an experiment.^54^

### ARC by Platform

ARC researchers predominantly explored the social media platform Facebook; nine out of 33 studies (27%)^47^,^50^,^53^,^54^,^68^,^75^,^80^,^84^,^87^ exclusively examined Facebook, three studies^58^,^66^,^88^ solely looked into Instagram, and two studies^46^,^51^ only investigated Twitter. Moreover, seven studies (21%)^48^,^59^,^62^,^63^,^67^,^69^,^78^ that explored multiple platforms all included both Facebook and Instagram; in addition to those two platforms, six studies (18%)^59^,^62^,^63^,^67^,^69^,^78^ also encompassed Snapchat, four studies (12%)^48^,^62^,^63^,^69^ included Twitter, two studies (6%)^67^,^78^ assessed WhatsApp, and a single study appraised TikTok, YouTube, and Reddit (3%).^62^ The remaining studies (36%)^52^,^55^–^57^,^60^,^61^,^64^,^72^,^73^,^76^,^86^,^89^ explored social media ARC more broadly.

### ARC Measures

Across the 33 studies that evaluated the effects of ARC use and alcohol outcomes among young adults, there was a high degree of variability in the measures used to assess ARC. Most commonly used were self-report assessments using either single items (64%)^46^,^47^,^57^–^63^,^66^–^69^,^72^,^73^,^75^,^76^,^78^,^86^,^88^,^89^ or multi-item measures created by the researchers for their respective studies (24%).^48^,^52^–^56^,^61^,^64^ A minority of studies (21%) examined content shared by participants by having researchers friend/follow participants and manually (or with the assistance of technology) extract and code ARC posts.^50^,^51^,^58^,^66^,^80^,^84^,^87^ This assessment of researcher-coded ARC posts was used alone or sometimes alongside self-report assessments. Three studies (9%) used two ARC assessment methods (single items and content analyses).^58^,^62^,^66^

There was inconsistency in what the single items were capturing, with most assessing frequency of exposure to ARC posts or posting ARC (39%).^46^,^47^,^57^–^59^,^62^,^63^,^66^,^72^,^73^,^76^,^78^,^88^ Of note, one study^60^ averaged frequency of exposure to multiple types of alcohol-related media, including print, movies, and social media. Most of the single items used dichotomous or Likert scale response options (15%)^57^,^61^,^68^,^69^,^86^ or aggregated the number/percentage of ARC posts that participants were exposed to or posted (12%).^60^,^67^,^75^,^89^ There was no one standardized or validated multi-item self-report measure used to assess ARC across the studies that were extracted. The scales that were developed included from seven to 13 items and were all unidimensional. Six studies (18%)^48^,^52^,^55^,^56^,^61^,^64^ assessed ARC without specifying a platform, using measures such as the Alcohol-related Social Media Use Index, Alcohol and Social Networking Practice Questionnaire, and Alcohol Social Networking Site Posting; a couple of studies^53^,^54^ focused on Facebook ARC, using the Facebook Alcohol Questionnaire or Alcohol-Related Facebook Activity Questionnaire. Only three studies^52^–^54^ reported performing an exploratory factor analysis (i.e., an approach used to assess specific factors without having a pre-existing theoretical structure) or principal components analysis (i.e., a method that includes correlated variables to limit the number of variables in the analysis) to examine the measurement structure; however, few details were included, suggesting that potential additional validation is needed. Of note, most studies using multi-item self-report measures (18%)^52^–^56^,^64^ included posting, exposure, and engagement in the same scale or would sometimes include other general measures of social media use such as number of friends/followers or time spent on social media. Only two studies using multi-item measures solely focused on ARC posting alone.^48^,^61^

### ARC Exposure and Drinking Outcomes

The research team elected to categorize studies based on whether they assessed exposure to ARC or posting ARC in connection to drinking outcomes. Studies that investigated both exposure and posting were reviewed separately to evaluate the combined effect on drinking outcomes.

Roughly one-third of all included studies^47^,^52^,^55^–^61^,^76^,^78^,^88^ examined exposure to ARC in relation to alcohol consumption; half of these studies also investigated alcohol-related problems as an outcome variable.^47^,^52^,^57^,^60^,^61^,^76^ Several studies^55^–^59^,^61^,^78^,^88^ examined pure relationships, defined as associations in which there were no conceptual overlaps between ARC predictor variables and drinking outcomes. All of these studies uncovered a positive, significant relationship—that is, increased ARC exposure was associated with increased alcohol use. One study also investigated the relationship between exposure to ARC and alcohol-related problems (e.g., missing work or classes, driving under the influence), but did not find a significant linkage.^57^ Additionally, one study also explored the linkage between engagement with ARC and drinking and found a positive, significant association.^55^

Several studies^47^,^52^,^60^,^76^ investigated relationships between ARC exposure and drinking and/or alcohol-related problems (e.g., injuries as a result of drinking, damage to a friendship, impulsive behavior, inability to stop drinking), but exhibited confounding issues that prevented disentangling the pure associations between these linkages. One longitudinal diary study^60^ calculated a mean score for the average exposure to ARC on social media in combination with other forms of alcohol-related media (e.g., movies, TV, radio) to predict heavy episodic drinking and related problems; this made it difficult to distinguish the effect of ARC solely related to social media. For two studies^47^,^76^ that utilized the total AUDIT score to assess alcohol outcomes, it was challenging to isolate the pure relationships between exposure to ARC and alcohol consumption or exposure to ARC and alcohol-related problems because the AUDIT includes items that assess both. Finally, one study^52^ combined measures of exposure to ARC and engagement with ARC into one predictor variable. Still, these studies found a positive, significant link between exposure to ARC and drinking and/or related problems.

### ARC Posting and Drinking Outcomes

Six out of 33 studies (18%) explored the association between posting ARC and alcohol consumption,^51^,^66^,^68^,^69^,^72^,^86^ three of which also examined associations with alcohol-related problems.^51^,^72^,^86^ All six studies reported positive, significant associations between posting ARC and drinking—that is, higher levels of ARC posting were associated with higher alcohol consumption levels.^51^,^66^,^68^,^69^,^72^,^86^ Two studies extrapolated specific characteristics of ARC posts (e.g., posting pictures of oneself drinking or references to alcohol) and examined those aspects separately in relation to drinking.^66^,^68^ In addition to exploring the link between posting ARC and drinking, one study also examined engagement with ARC in relation to consumption and uncovered a positive, significant association.^69^ The studies that also assessed association between posting ARC and alcohol-related problems all found a positive, significant relationship.^51^,^72^,^86^ One additional study that only examined the relationship between ARC posting and alcohol-related problems also found a positive, significant linkage.^48^

### ARC Posting and Exposure and Drinking Outcomes

Fourteen out of 33 studies (42%)^46^,^50^,^53^,^54^,^62^–^64^,^67^,^73^,^75^,^80^,^84^,^87^,^89^ investigated both exposure to ARC and posting ARC in relation to alcohol consumption. In terms of pure associations, seven studies^53^,^62^,^63^,^67^,^73^,^75^,^89^ found positive, significant linkages between posting ARC and drinking. For exposure to ARC, four studies^46^,^63^,^73^,^89^ reported positive, significant associations with drinking, while three did not.^62^,^67^,^75^ Two studies^54^,^87^ also examined associations with alcohol-related problems and found positive, significant relationships between both exposure to ARC and posting ARC and such problems.

There were complications in extrapolating the pure associations between ARC exposure/posting and drinking/alcohol-related problems in eight studies.^46^,^50^,^53^,^54^,^64^,^80^,^84^,^87^ A few studies^53^,^54^,^87^ utilized the AUDIT total score to assess alcohol outcomes which, as previously mentioned, assesses both consumption and alcohol-related problems simultaneously. Additionally, these studies, along with a few others,^50^,^53^,^54^,^64^,^80^,^84^,^87^ assessed ARC posting by examining Facebook “wall posts,” which may represent both ARC posting and exposure. Thus, posts to a person’s wall could be made by either the profile owner themselves (which would constitute posting ARC) or by another person (which would be classified as exposure to ARC). Finally, in one study^46^ it was challenging to ascertain the unique contribution of ARC posting to drinking because the study combined both posting ARC and following pro-alcohol accounts on Twitter (i.e., engagement with ARC) to assess “active exposure.”

### Mediators and Moderators of ARC–Drinking Associations

Of the 33 studies explored, seven (21%) examined mediators or moderators of the association between ARC (posting, exposure, or engagement) and alcohol consumption.^48^,^58^,^59^,^63^,^69^,^78^,^88^ In a cross-sectional study, Vranken et al.^78^ found that friends’ pro-drinking social norms, measured via descriptive norms (i.e., perceived prevalence of drinking within a specific population, such as friends) and injunctive norms (i.e., perceived approval of drinking within a specific population) exerted positive indirect effects on associations between Facebook and Snapchat ARC exposure and alcohol use. In contrast, personal pro-drinking attitudes significantly positively mediated associations between Instagram ARC exposure and drinking. LaBrie, Trager, et al.^58^ determined that self-reported (i.e., subjective) ARC exposure and descriptive norms were significant positive sequential mediators of the association between objective ARC exposure (i.e., measured via systematic sampling of participants’ Instagram feeds and recording the amount of time spent on the platform) and later drinking. Thompson and Romo^48^ reported that ARC posting exerted a positive cross-sectional indirect effect on the associations between alcohol identity, adherence to social norms (popularity, peer pressure), and alcohol-related problems. Using moderated serial mediation, Alhabash et al.^69^ found that when participants did not engage with ARC posted by others, the association between their own ARC posting and drinking was mediated by peers’ and close friends’ norms. However, when participants did engage with ARC posted by others, norms no longer mediated the association between self-posting ARC and personal drinking.

Several studies assessed whether biological sex mediated associations between ARC posting/exposure and alcohol consumption. Davis et al.^63^ analyzed the role of biological sex in moderating associations between ARC posting, ARC exposure, and drinking over time. For both males and females, only ARC posting was associated with greater drinking over time. In contrast, the link between ARC exposure and drinking was only significant at certain times of the school year, which differed between males and females (pre-matriculation to college for males and in the first semester of college for females). Boyle et al.^59^ reported that descriptive norms for peak drinks (i.e., perceptions of peak number of drinks consumed by a typical student) significantly and positively mediated the association between ARC exposure and later drinking for males only. Finally, LaBrie, Boyle, et al.^88^ explored ARC exposure as a mediator rather than a predictor and found that it significantly and positively mediated associations between having a Finsta (i.e., “fake Instagram,” which normally refers to a secret account created for the purpose of engaging exclusively with select others^96^) and later drinking only for males.

## Discussion

### Summary of Evidence and Methodological Issues

The findings of this scoping review of the literature revealed a lack of consistent operationalizations for exposure to ARC, posting ARC, and engagement with ARC (see Table 2). In fact, some studies created their own study-specific terms related to alcohol-related social media usage (e.g., Cabrera-Nguyen et al.^46^ grouped posting ARC and following pro-alcohol accounts on Twitter under the umbrella term “active exposure”); thus, it was difficult to relate these findings to the broader literature. Furthermore, there are no empirically validated ARC measures, and researchers often devised their own ways of assessing exposure to ARC, posting ARC, and/or engagement with ARC.

Moving forward, a consensus on ARC definitions and the use of standardized ARC measures would make findings more generalizable across studies. For example, researchers could use a modified version of the well-validated Alcohol Timeline Followback assessment,^97^ which uses a calendar format to assess participants’ drinking over a particular time period. However, instead of using traditional anchor dates (i.e., holidays, birthdays, anniversaries, stressful events) from which to recall their drinking, participants could use their social media posts as a way to remember their drinking habits. This would allow researchers to not only better assess the number of participants’ ARC posts, but it may also improve participants’ recollection of their drinking because social media posts may serve as a digital diary of an individual’s activities over a specified period.^98^

Despite the lack of consistent operationalizations of ARC constructs and empirically validated ARC measures, as well as other potential confounding issues, studies that explored the relationship between posting ARC and drinking unanimously found a positive, significant association.^46^,^50^,^51^,^53^,^54^,^62^–^64^,^66^–^69^,^72^,^73^,^75^,^80^,^84^,^86^,^89^ The findings were less clear with regard to the relationship between exposure to ARC and drinking. However, irrespective of whether there were possible confounds, most of the studies that examined exposure to ARC and drinking (73%) found a positive, significant association.^46^,^47^,^50^,^52^,^54^–^64^,^73^,^75^,^76^,^78^,^80^,^84^,^87^–^89^ Moreover, the bulk of the studies that examined both ARC exposure and posting, and which did not have confounding issues,^62^,^63^,^73^,^75^,^89^ uncovered a stronger association between ARC posting and drinking than between ARC exposure and drinking. This finding suggests that when including both aspects of ARC in the models, posting ARC may explain more of the variance in predicting drinking than exposure to ARC.

The pattern of the associations between ARC exposure and posting and alcohol-related problems mirrors the ones with consumption as the outcome variable. All studies that investigated the relationship between posting ARC and alcohol-related problems discovered a positive, significant association.^48^,^51^,^53^,^54^,^72^,^86^ Almost all studies that explored exposure to ARC in relation to alcohol-related problems, irrespective of confounding issues, also revealed a positive, significant linkage.^47^,^52^–^54^,^60^,^61^,^76^,^87^ It is possible that posting ARC indicates that the person has integrated a drinking identity into their self-concept, leading them to drink more and experience greater problems. In fact, a recent study analyzing Facebook posts found that the language contained in social media posts may be reasonably precise in helping to diagnose individuals at risk for alcohol-related problems and alcohol use disorder;^99^ thus, the potential drinking identities that individuals display via social media may be used in prevention efforts to identify those at risk. Furthermore, ARC posts may reflect the person’s belief that their peers approve of drinking and posting ARC. In fact, perceptions of peer drinking norms are one of the strongest predictors of young people’s drinking behaviors and problems.^100^

Only a handful of studies (12%)^46^,^52^,^55^,^69^ examined ARC engagement as well as posting and exposure in relation to drinking and alcohol problems, making it difficult to draw any definitive conclusions about these associations. However, engagement with ARC may be an important concept for literature to continue to explore concurrently with exposure to and posting of ARC. As previously mentioned, young people’s engagement with others’ ARC content often provides positive validation for that content, which may both encourage the person to continue posting ARC and influence others within the network to post ARC to receive similar social affirmation.^101^

The authors of this scoping review also noted several inconsistencies in inclusion criteria across the 33 studies. First, only a handful of studies specifically required that participants met drinking criteria (12%),^48^,^60^,^61^,^86^ and less than half of the studies (39%)^48^,^54^,^57^,^58^,^63^,^66^,^67^,^72^,^80^,^86^–^89^ explicitly mentioned social media inclusion criteria. Moreover, only one study explicitly specified criteria for heavy drinking,^60^ and most samples did not appear to include participants who drank heavily (55%).^46^,^47^,^53^,^54^,^58^,^59^,^61^–^63^,^66^–^68^,^72^,^80^,^84^,^87^–^89^ Given that populations at risk for alcohol misuse presumably are the main targets for interventions pertaining to ARC and drinking, further research should consider examining ARC in relation to populations who drink heavily. Moreover, stating inclusion criteria more clearly and consistently could help other researchers better extrapolate how findings relate to specific populations of social media users or people who drink. This would aid in the replicability of the findings.

Other gaps in the literature include the lack of objective measures of ARC and a dearth of variation in study designs (nearly 70% of studies employed a cross-sectional design^46^–^48^,^51^–^53^,^55^–^57^,^61^,^62^,^64^,^66^,^68^,^69^,^73^,^75^,^76^,^78^,^80^,^86^,^89^). Both self-report measures and cross-sectional designs are prone to self-report^102^ and recall bias,^103^ respectively. Thus, researchers may want to consider more objective measures, preferably in combination with subjective measures because people’s ARC-related perceptions might be more indicative of their drinking behaviors than actual ARC behaviors. Additionally, future research may benefit from use of other study methodologies such as ecological momentary assessments, which reduce recall bias, allow for inferences regarding temporal order, and disentangle between-person (i.e., across individuals) and within-person associations.^103^ The implementation of these measures would help the field move forward by improving replicability, interpretability, and reliability of findings; furthermore, these approaches could help to minimize bias and allow the findings to be more easily synthesized in the future.

### Future Directions

A noteworthy finding was that the vast majority of the studies included in this scoping review were composed of White (79%),^46^,^48^,^50^–^55^,^57^–^64^,^68^,^69^,^72^,^75^,^86^–^89^ female (88%)^46^–^48^,^50^–^55^,^57^–^64^,^66^,^68^,^69^,^72^,^75^,^76^,^78^,^80^,^84^,^86^–^89^ college students (82%)^47^,^48^,^50^,^52^–^54^,^56^–^61^,^63^,^64^,^66^,^68^,^72^,^73^,^75^,^76^,^80^,^84^,^86^–^91^ living in the United States (73%).^46^,^48^,^50^–^52^–^54^,^57^–^64^,^68^,^69^,^72^,^80^,^84^–^89^ The dearth of literature examining the relationship between ARC and drinking among other subpopulations hinders a comprehensive understanding of how different demographic groups perceive, engage with, and are potentially influenced by ARC on social media. Consequently, tailored interventions and policies aimed at mitigating negative outcomes associated with alcohol consumption through digital media remain underexplored and insufficiently informed by diverse perspectives and experiences.

Most of the literature reviewed in this scoping review indicated that ARC might be associated with young people drinking more. However, it is possible that interacting with ARC on social media related to sober-curious movements (e.g., Dry January^104^) could instigate positive behavioral change. For instance, a scoping review found that temporary abstinence challenge participants reported not only reduced alcohol use but also other health benefits such as weight loss and improvements in sleep.^105^ Still, the studies reviewed did not focus on participants’ posting and engagement with sober curious social media content. Future research should examine whether posting of or engagement with sober-curious content could facilitate short or long-term reductions in young people’s alcohol use.

Although evidence suggests ARC posting and exposure may negatively impact young people’s drinking, there currently is still a paucity of interventions designed to directly address this issue.^106^–^108^ One qualitative study that asked participants to rank possible intervention strategies reported that young people considered automated warnings (i.e., utilizing machine learning to flag depictions of alcohol and having users confirm that they wish to upload such content) to be the most effective tactic.^106^ The authors of that study acknowledged that this type of intervention may not be feasible given that social media platforms would have to allow researchers to access their application programming interfaces in order to implement the machine learning aspect of the intervention. However, implementing the suggestions outlined in this scoping review in the future might ensure that researchers can harness the information derived from the literature and devise ARC-related interventions for populations at risk for problems with alcohol or alcohol use disorder.

While most of the studies reviewed here solely explored the associations between ARC posting/exposure and alcohol-related outcomes (albeit in different ways), less than one-quarter of articles investigated possible mediators and moderators of these associations (21%).^48^,^58^,^59^,^63^,^69^,^78^,^88^ Moreover, nearly 40% of studies examined did not mention the use of a particular theory to explain the associations.^46^–^58^ Additional research may want to examine the individual differences, social contextual factors, and environmental factors that may be driving these effects; such research could help identify which young people are more susceptible to ARC and why. For instance, examining these factors in relation to young people’s motivations for constructing their self-identity around ARC may help enable clinicians to redirect these behaviors. Moreover, while one study found that drinking prospectively predicted posting of ARC,^109^ additional research could further investigate the temporal order and/or bidirectionality of the linkage between ARC and alcohol use. Relatedly, it is still unclear whether the cycle of posting ARC and drinking is due to homogeneity (i.e., people who drink more and post more ARC tend to associate with like-minded people) and/or whether it is because people become entrenched in a group that tends to drink more and post more, and consequently adopt similar behaviors. Disentangling these associations may be key in prevention efforts.

### Proposed Theoretical Model

**Figure 2:**
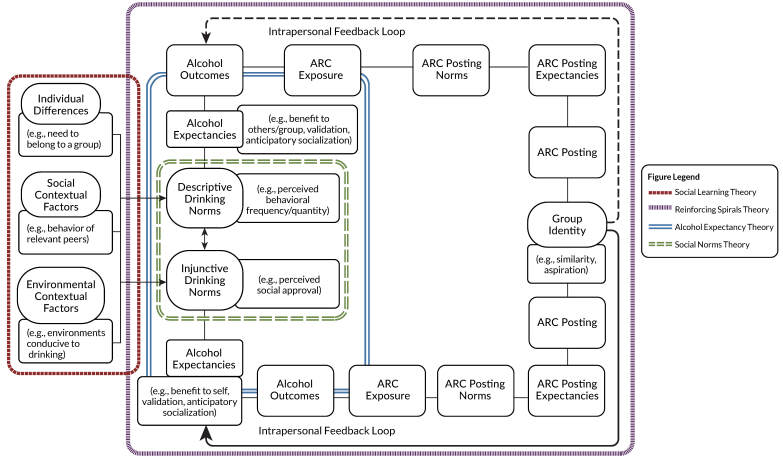
The dual-feedback loop drinking and ARC model. The model incorporates aspects of social learning theory (maroon, thick dashed line), reinforcing spirals theory (purple, thin dashed line), alcohol expectancy theory (blue, solid double line), and social norms theory (green, double dashed line) to explain how and why young people are attracted to join a group that encourages alcohol misuse and ARC posting via homogeneity in drinking behavior and/or aspirations to be accepted by the group that drinks heavily. *Note:* ARC, alcohol-related content.

This scoping review identified several gaps in the literature, including the need for a comprehensive theoretical framework to advance and propose future directions within the field. Consequently, the authors propose a Dual-Feedback Loop Drinking and ARC Model that encompasses a combination of previously established theories commonly utilized within the field and posits directional associations in terms of drinking and posting. Furthermore, the model explains how and why an individual is attracted to join a group that encourages alcohol misuse and ARC posting—for example, via homogeneity in drinking behaviors and/or via individual differences, such as aspirations to fit in with a group that drinks heavily. Specifically, this conceptual model incorporates social learning,^17^ social norms,^20^,^21^,^65^ alcohol expectancy,^70^ and reinforcing spirals theories^27^ (see Figure 2) to explain both individual and group dynamics that contribute to heavy drinking and frequent ARC posting.

First, certain individual differences (such as already drinking heavily or a strong need to belong) in combination with social contextual factors (such as wanting to join a sorority) and environmental contextual factors (such as attending a college with a reputation for partying) contribute to the development of an individual’s descriptive and injunctive drinking norms. For example, a college freshman who undergoes the sorority recruitment process may witness older students from her desired sorority engaging in heavy alcohol use. This, in turn, leads her to form descriptive norms about the drinking behaviors of sorority members (e.g., perceiving that members drink in large quantities) as well as to derive injunctive norms (e.g., perceiving that most members of the sorority approve of such drinking).

From these norms, an intrapersonal feedback loop may form. She may develop alcohol expectancies such that she believes that if she engages in similar drinking, it may increase her likelihood of being accepted by the sorority. Consequently, after she initiates heavy drinking in front of sorority members, they might begin adding her on social media platforms where they post ARC. This exposure to the sorority members’ ARC not only reinforces drinking norms, but also leads her to develop descriptive and injunctive norms regarding ARC posting behaviors, which, likewise, inform ARC posting expectancies (i.e., she believes that if she engages in similar ARC posting, she will receive the same sort of social validation in the form of engagement).

Moreover, she may conclude that posting her own ARC will solidify her status as an in-group member. As a result, she begins to post ARC of her own and receives the validation she expects from group members, reinforcing her drinking and ARC posting norms and expectancies. If she perceives that her efforts to assimilate into the group by emulating members’ drinking and posting behaviors are rewarded, she may begin to integrate her perception of the sorority group drinking identity into her own identity (i.e., group identity via aspirations to assimilate). Thus, an intrapersonal feedback loop is initiated whereby she continues or possibly even escalates her own drinking and posting behaviors to affirm to herself that she is part of the group.

At the same time, sorority members may be involved in social gatherings in which most members engage in drinking, thereby contributing to perceptions of collective drinking norms among the group members. These collective drinking norms may lead to positive alcohol expectancies at the group level, encouraging sorority members to engage in more alcohol use. In addition, senior members of the sorority may post ARC and receive validation from other sorority members. If junior members of the sorority are exposed to this ARC and the popularity of the posts, they may develop collective ARC posting expectancies (i.e., if they post their own ARC, they will be similarly validated by fellow sorority members). This, in turn, encourages junior members to engage in ARC posting, which fosters group cohesion and reinforces the sorority’s group identity as people who drink heavily and frequently post ARC. As the strength of the sorority’s collective drinking and posting identities increases, it instigates an interpersonal feedback loop through which new members are assimilated into this culture of intoxication.^29^ Finally, the

combination of these interpersonal and intrapersonal feedback loops may contribute to the overinflation of drinking norms and subsequent cyclical increases in drinking among sorority group members.

### Limitations of the Review

Although this scoping review provides a comprehensive summary of the literature, some limitations should be considered. First, some studies were eliminated during the screening process because of the stringent age and study design criteria (e.g., no qualitative studies). Second, although the authors cast broad parameters with respect to criteria related to sample populations (i.e., young adults ages 18 to 30), as mentioned previously, the bulk of study participants consisted of White, female college students residing in the United States; thus, this review may not be generalizable to all young adults and racial/ethnic minorities, as well as to those from countries other than the United States.

### Conclusions

The aim of this scoping review was to examine how researchers have approached the complex relationships between young people’s ARC use and drinking outcomes. In the digital age, young people increasingly rely on social media as a conduit for communicating both their values and their identities surrounding alcohol;^16^,^110^ in doing so, they may be socially influencing other people within their networks to also post ARC and drink, potentially leading to social contagion of heavy drinking within a network. Overall, this scoping review highlights that to address this critical issue, it is imperative that researchers come to a consensus on the operationalizations and standardizations of ARC measurements (i.e., best practices regarding objective assessments and well-validated measurement tools for subjective assessments). Furthermore, more research is needed on individual differences (e.g., personality traits, motivations) in relation to these associations. This might allow researchers to better identify the characteristics of at-risk populations and tailor interventions for these vulnerable groups.

**KEY TAKEAWAYS**

- The results of this scoping review confirm strong positive links between posting alcohol-related content (ARC) and drinking and alcohol-related problems.
- The findings in this review underscore the need for operationalization and standardized measures related to ARC.
- The authors propose a theoretical model—the Dual-Feedback Loop Drinking and ARC Model—that may serve as a blueprint for future interventions targeting young adults.

## Figures and Tables

**Table 1:** Search Strategies by Database

Database	Search Strategy (all searches limited to English language, published 2006 to present)	No. of Results Retrieved
PubMed	Original search: May 30, 2023 (“Facebook*”[tiab] OR “Flickr*”[tiab] OR “Instagram*”[tiab] OR “MySpace*”[tiab] OR “Online Social Networking”[MeSH] OR “Pinterest*”[tiab] OR “Reddit*”[tiab] OR “Snapchat*”[tiab] OR “Social Media”[MeSH] OR “Social media*”[tiab] OR “social networking platform*”[tiab] OR “social networking site*”[tiab] OR “social networking website*”[tiab] OR “social networking web site*”[tiab] OR “social platform*”[tiab] OR “TikTok*”[tiab] OR “Tik Tok*”[tiab] OR “Tumblr*”[tiab] OR “Twitter*”[tiab] OR “YouTube*”[tiab] OR “Bebo*”[tiab] OR “BeReal*”[tiab] OR “Sina Weibo*”[tiab] OR “Telegram*”[tiab] OR “Twitch*”[tiab] OR “Vine”[tiab] OR “VK*”[tiab] OR “WeChat*”[tiab] OR “WhatsApp*”[tiab] OR “YikYak*”[tiab]) AND (“Alcoholic Beverages”[MeSH] OR “Alcohol Drinking”[MeSH] OR “alcohol*”[tiab] OR “Alcoholism”[MeSH] OR “Alcohol-Related Disorders”[MeSH] OR “Binge Drink*”[tiab] OR “Underage Drink*”[tiab]) AND (“Adolescent”[MeSH] OR “Adult”[MeSH:NoExp] OR “young adult*”[tiab] OR “Young Adult”[MeSH])	539
	Repeat search: November 1, 2023	53
CINAHL	Original search: May 30, 2023 ((MH “Online Social Networking+”) OR (MH “Social Media+”) OR “Bebo*” OR “BeReal*” OR “Facebook*” OR “Flickr*” OR “Instagram*” OR “MySpace*” OR “Pinterest*” OR “Reddit*” OR “Sina Weibo*” OR “Snapchat*” OR “Social media*” OR “social networking platform*” OR “social networking site*” OR “social networking web site*” OR “social networking website*” OR “social platform*” OR “Telegram*” OR “Tik Tok*” OR “TikTok*” OR “Tumblr*” OR “Twitch*” OR “Twitter*” OR “Vine” OR “VK*” OR “WeChat*” OR “WhatsApp*” OR “YikYak*” OR “YouTube*”) AND ((MH “Alcoholic Beverages+”) OR (MH “Alcohol Drinking+”) OR (MH “Alcoholism+”) OR (MH “Alcohol-Related Disorders+”) OR “alcohol-related” OR “alcohol*” OR “Binge Drink*” OR “Underage Drink*”) AND ((MH “Adolescent+”) OR (MH “Adult”) OR (MH “Young Adult+”) OR “young adult*”)	350
	Repeat search: November 1, 2023	32
APA PsycInfo (EBSCOhost)	Original search: May 30, 2023 ((MH “Online Social Networking+”) OR (MH “Social Media+”) OR “Bebo*” OR “BeReal*” OR “Facebook*” OR “Flickr*” OR “Instagram*” OR “MySpace*” OR “Pinterest*” OR “Reddit*” OR “Sina Weibo*” OR “Snapchat*” OR “Social media*” OR “social networking platform*” OR “social networking site*” OR “social networking web site*” OR “social networking website*” OR “social platform*” OR “Telegram*” OR “Tik Tok*” OR “TikTok*” OR “Tumblr*” OR “Twitch*” OR “Twitter*” OR “Vine” OR “VK*” OR “WeChat*” OR “WhatsApp*” OR “YikYak*” OR “YouTube*”) AND ((MH “Alcoholic Beverages+”) OR (MH “Alcohol Drinking+”) OR (MH “Alcoholism+”) OR (MH “Alcohol-Related Disorders+”) OR “alcohol*” OR “Binge Drink*” OR “Underage Drink*”) AND ((MH “Adolescent+”) OR (MH “Adult”) OR (MH “Young Adult+”) OR “young adult*”)	493
	Repeat search: November 1, 2023	34
Scopus	Original search: May 30, 2023 (TITLE-ABS-KEY(“Facebook*”) OR TITLE-ABS-KEY(“Flickr*”) OR TITLE-ABS-KEY(“Instagram*”) OR TITLE-ABS-KEY(“MySpace*”) OR INDEXTERMS(“Online Social Networking”) OR TITLE-ABS-KEY(“Pinterest*”) OR TITLE-ABS-KEY(“Reddit*”) OR TITLE-ABS-KEY(“Snapchat*”) OR INDEXTERMS(“Social Media”) OR TITLE-ABS-KEY(“Social media*”) OR TITLE-ABS-KEY(“social networking platform*”) OR TITLE-ABS-KEY(“social networking site*”) OR TITLE-ABS-KEY(“social networking website*”) OR TITLE-ABS-KEY(“social networking web site*”) OR TITLE-ABS-KEY(“social platform*”) OR TITLE-ABS-KEY(“TikTok*”) OR TITLE-ABS-KEY(“Tik Tok*”) OR TITLE-ABS-KEY(“Tumblr*”) OR TITLE-ABS-KEY(“Twitter*”) OR TITLE-ABS-KEY(“YouTube*”) OR TITLE-ABS-KEY(“Bebo*”) OR TITLE-ABS-KEY(“BeReal*”) OR TITLE-ABS-KEY(“Sina Weibo*”) OR TITLE-ABS-KEY(“Telegram*”) OR TITLE-ABS-KEY(“Twitch*”) OR TITLE-ABS-KEY(“Vine”) OR TITLE-ABS-KEY(“VK*”) OR TITLE-ABS-KEY(“WeChat*”) OR TITLE-ABS-KEY(“WhatsApp*”) OR TITLE-ABS-KEY(“YikYak*”)) AND (INDEXTERMS(“Alcoholic Beverages”) OR INDEXTERMS(“Alcohol Drinking”) OR TITLE-ABS-KEY(“alcoholic”) OR TITLE-ABS-KEY(“alcohol-related”) OR INDEXTERMS(“Alcoholism”) OR INDEXTERMS(“Alcohol-Related Disorders”) OR INDEXTERMS(“Binge Drink*”) OR TITLE-ABS-KEY(“Underage Drink*”)) AND (INDEXTERMS(“Adolescent”) OR INDEXTERMS(“Adult”) OR TITLE-ABS-KEY(“young adult*”) OR INDEXTERMS(“Young Adult”))	210
	Repeat search: November 1, 2023	50

*Note*.

* search for variant word endings; “ “ = search for exact phrases;+ = search for narrower subject headings in CINAHL; [MeSH], MH, & (INDEXTERMS) = search subject headings within each database; [tiab] & (TITLE-ABS-KEY) = search titles, abstracts, and keywords

**Appendix 1:** Sample Characteristics, Designs, and Methods of All Articles Included in the Scoping Review

Author, Year Country*	Sample Size and Drinking Level**	Design	Demographics	ARC Predictors	Alcohol Outcomes†
Erevik et al., 2018^76^ *Norway*	11,236 college students who drink heavily	Cross-sectional, survey	63% female, 37% male	Posting and exposure: Frequency of self-posting and exposure of ARC depicting positive consequences of drinking and negative consequences of drinking (separate questions) from “never” to “daily or almost daily”	AUDIT (+)
Moreno et al., 2012^87^ *United States*	224 college students who do not drink heavily	Cross-sectional, survey, researcher-coded posts	55% female, 45% male 68% White, 17% Asian, 4% other, 8% multiracial	Posting: Profile evaluation by researchers to identify ARC (general alcohol use, intoxication)	AUDIT (+)
Oliva et al., 2018^47^ *Mexico*	110 college students who do not drink heavily	Cross-sectional, survey	58% female, 42% male	Exposure: Frequency of seeing photo ARC on Facebook in the past 30 days (no response options reported)	AUDIT (+)
Critchlow et al., 2017^55^ *United Kingdom*	405 non-college young adults who drink heavily	Cross-sectional, survey	72% female, 28% male 66% White, 34% other	Exposure and engagement: Awareness/exposure to ARC (yes/no) and interaction with ARC (yes/no). Examples included status updates, photos, messages, and videos.	AUDIT-C (+ for exposure and engagement)
Miller et al., 2014^75^ *Australia*	134 college students who drink heavily	Cross-sectional, survey	100% female 61% White, 33% Asian, 5% other	Posting and exposure: Percentage of own and friends’ posts that are ARC. Response options ranged from 0% to 100% in 25% increments.	AUDIT-C (+ for posting, NS for exposure)
Vanherle et al., 2022^67^ *Belgium*	337 college students who do not drink heavily	Daily diary	50% female, 50% male	Posting and exposure: Frequency of ARC posted in the past 24 hours from 0 = none to 4 = more than 10; frequency of ARC viewed in the past 24 hours from 0 = none to 4 = a lot	Modified one question from the AUDIT-C (+)
Atusingwize et al., 2022^73^ *Uganda*	996 college students who may drink heavily	Cross-sectional	46% female, 54% male	Posting and exposure: Frequency of self-posting ARC and frequency of viewing but not engaging with ARC were assessed with response options from “never” to “10 or more times a day.” These were then recoded into dichotomous responses (i.e., yes/no).	Modified one question from the AUDIT (Frequency) (+ for posting and exposure)
Vranken et al., 2020^78^ *Belgium*	905 non-college young adults who drink heavily	Cross-sectional, survey	73% female, 27% male	Exposure: Frequency of exposure to pictures, videos, or text messages referring to alcohol from 0 = never to 6 = several times a day	Modified two items from AUDIT (Consumption and frequency) (+)
Boyle et al., 2016^59^ *United States*	412 college students who do not drink heavily	Longitudinal, survey	64% female, 36% male 54% White, 11% Asian, 9% Black, 4% multiracial or other 22% Hispanic, 78% non-Hispanic	Exposure: Frequency of seeing text or photo peer ARC (response options 0 = never to 4 = always)	DDQ (+)
Davis et al., 2021^63^ *United States*	320 college students who do not drink heavily	Longitudinal, survey	61% female, 39% male 46% White, 10% Black, 15% Asian, 17% Hispanic, 8% multiethnic, 1% other	Posting and exposure: Past-month frequency of posting text or photo ARC (options from 0 = never to 4 = always). Past month frequency of exposure to ARC was also assessed (same response options as posting)	DDQ (+ for posting and exposure)
LaBrie et al., 2023^88^ *United States*	296 college students who do not drink heavily	Longitudinal, survey	63% female, 37% male 47% White, 8% Black, 16% Asian, 1% Native Hawaiian, 9% multiracial or other 18% Hispanic, 82% non-Hispanic	Exposure: Frequency of exposure to ARC with response options for from 0 = never to 4 = always	DDQ (+)
LaBrie et al., 2021^58^ *United States*	309 college students who do not drink heavily	Longitudinal, survey, researcher-coded posts	62% female, 38% male 47% White, 10% Black, 16% Asian, 10% multiracial or other 18% Hispanic, 83% non-Hispanic	Exposure: ARC exposure was captured by using a Python macro that randomly sampled posts from participants’ Instagram profiles and coded them as alcohol or not; frequency of ARC exposure (e.g., posts depicting alcohol consumption) was determined with response options from 1 = never to 5 = always	DDQ (+)
Steers et al., 2021^89^ *United States*	780 college students who do not drink heavily	Cross-sectional	68% female, 32% male 49% White, 11% Black, 30% Asian, 4% American Indian/Alaska Native, 1% Native Hawaiian/Pacific Islander, 5% other	Posting and exposure: frequency of self-posting and exposure to ARC in the past 3 months from using modified DDQ to reflect the number of ARC posts viewed on each day the week	DDQ (+ for posting and exposure)
Rodriguez et al., 2016^80^ *United States*	109 college students who do not drink heavily	Cross-sectional, survey, researcher-coded posts	88% female, 12% male 47% White, 11% Black, 17% Asian, 6% multiethnic, 19% other 32% Hispanic, 68% non-Hispanic	Posting: Profile evaluation by researchers to identify ARC in most recent 100 posts; posts then were summed	DDQ—drinks per week (+) QFI—Drinking frequency, typical quantity, and number of drinks (+)
Strowger et al., 2022^61^ *United States*	130 college students who do not drink heavily	Cross-sectional, survey	86% female, 12% male 57% White, 23% Black, 4% Asian, 12% more than one race, 5% other 88% non-Hispanic, 11% Hispanic	Exposure: Participants were asked to list up to 10 people who had been important in their lives in the past year then were asked if each person had shared ARC (yes/no). These responses were then averaged	DDQ (+) BYAACQ (NS)
Strowger et al., 2023^57^ *United States*	528 college students who drink heavily	Cross-sectional, survey	24% male, 75% female 27% Black, 4% Asian/Asian American, 69% White, 1% Native American or Pacific Islander, 3% other 9% Hispanic or Latinx, 91% non-Hispanic or Latinx	Exposure: Influencer post ARC (yes/no) and frequency of post ARC from 1 = never to 7 = daily or almost daily, whether influencer was an “actor, musician, professional athlete, politician, everyday person (ex. micro celebrity) or other” (select all that apply) and social media platforms they posted ARC on (select all that apply)	DDQ (+) BYAACQ (NS)
Trager et al., 2023^72^ *United States*	319 college students who do not drink heavily	Longitudinal, survey	62% female, 38% male 59% White, 16% Asian, 11% Black/African American, 13% multiracial/other 21% Hispanic, 79% non-Hispanic	Posting: Frequency of posting about partying, clubbing, going out; alcohol, getting drunk, being hungover; and marijuana, pot paraphernalia, getting high from 0 = never to 4 = always	DDQ (+) BYAACQ (+)
Graupensperger et al., 2023^60^ *United States*	201 college students who drink heavily	Longitudinal, daily diary	64% female, 36% male 55% White non-Hispanic, 17% Asian non-Hispanic, 11% other non-Hispanic (e.g., Black/African American non-Hispanic, multiracial non-Hispanic)	Exposure: Frequency of exposure to ARC in a typical week from 0 = none to 4 = many	Heavy episodic drinking frequency in the past 2 weeks (+) BYAACQ (+)
Litt et al., 2018^51^ *United States*	186 non-college young adults who may drink heavily	Cross-sectional, survey, researcher-coded posts	54% female, 46% male 49% White, 21% Black, 13% Asian, 1% American Indian or Alaska Native, 10% more than one race, 7% other 15% Hispanic, 85% non-Hispanic	Posting: Profile evaluation by researchers to identify ARC on Twitter	DDQ (+) YAACQ (+) AUDIT (+)
Westgate et al., 2014^53^ *United States*	1,099 college students who do not drink heavily	Cross-sectional, survey	41% male, 59% female 59% White, 27% Asian, 8% biracial or multiracial; the remaining 6% Black/African American, American Indian/Alaska Native, Native Hawaiian/Other Pacific Islander, unknown, or declined to answer	Posting and exposure: FAQ	DDQ (+ for posting, NS for exposure) RAPI (+ for posting, NS for exposure) AUDIT (+ for posting and exposure)
Thompson & Romo, 2016^48^ *United States*	364 college students who drink heavily	Cross-sectional, survey	61% female, 39% male 94% White, 4% Black, 2% Hispanic, 1% American Indian, 1% Asian, 2% other	Posting: Scale created by researchers assessed frequency of ARC posting (e.g., “I share what I am drinking on social networking sites”) from 1 = never to 7 = a great deal	RAPI (+)
Ward et al., 2022^86^ *United States*	1,063 college students who drink heavily	Cross-sectional, survey	66% female, 34% male 48% White, 11% Black, 27% Asian 34% Hispanic, 66% non-Hispanic	Posting: Participants were asked whether they posted ARC from 1 = definitely yes to 5 = definitely not	Number of drinks consumed during a peak drinking occasion in the past 30 days (+) Number of times typically consumed alcohol in the past 3 months (+) RAPI (+)
Moreno et al., 2013^50^ *United States*	66 college students who drink heavily	Longitudinal, survey, researcher-coded posts	68% female, 32% male 96% White, 4% non-White	Posting: Profile evaluation by researchers to identify ARC (general alcohol use, alcohol use during Mifflin [local holiday])	TLFB (+)
D’Angelo et al., 2014^84^ *United States*	312 college students who do not drink heavily	Longitudinal, survey, researcher-coded posts	57% female, 43% male	Posting: Profile evaluation by researchers to identify ARC	TLFB (+)
Marczinski et al., 2016^54^ *United States*	146 college students who do not drink heavily	Experiment, survey	58% female, 42% male 81% White, 12% Black, 2% Asian, 6% other 2% Hispanic, 98% non-Hispanic	Posting and exposure: Alcohol-related Facebook Activity Questionnaire^54^	TLFB (+) AUDIT (+) PDHQ (+)
Malechwanzi et al., 2022^56^ *Kenya*	836 college students who may drink heavily	Cross-sectional, survey	43% female, 57% male	Exposure: Number of posts appear in feed daily from 0–1 to 4–5, frequency of exposure (e.g., alcohol brand ad) from 1 = never to 5 = very often	TWEAK (+)
Alhabash et al., 2021^69^ *United States*	525 non-college young adults who drink heavily	Cross-sectional, survey	50% female, 50% male 71% White, 13% Black, 5% Asian, 1% American Indian/Alaska Native, 3% other 14% Hispanic, 86% non-Hispanic	Posting and engagement: Dichotomous questions (posting about and interacting with ARC on the platform most heavily used during Halloween)	Self-report # of drinks consumed on Halloween on scale from 0 to 25 (+ for posting and engagement)
Glassman, 2012^68^ *United States*	445 college students who do not drink heavily	Cross-sectional, survey	60% female, 40% male 76% White, 11% Black, 4% Asian/Pacific Islander, 2% Hispanic, 1% American Indian/Alaska Native, 5% other	Posting: Dichotomous question assessing self-posting of ARC	Average number of drinks consumed in a week and frequency of engagement in heavy episodic drinking in the past 2 weeks [whole numbers required for responses] (+)
Stoddard et al., 2012^64^ *United States*	3,448 college students who may drink heavily	Cross-sectional, survey	48% female, 52% male 70% White, 5% Black, 12% Asian/Pacific Islander, 9% Hispanic/Latino, 1% Native American, 1% other, 2% multiracial	Posting and exposure: Frequency of self-posting and exposure to ARC were assessed with five items. Response options ranged from 0 = none to 4 = almost all	Frequency of alcohol use in the past 30 days was assessed from 0 = never to 6 = more than once a day (+)
Hoffman et al., 2014^52^ *United States*	737 college students who may drink heavily	Cross-sectional, survey	68% female, 32% male 76% White, 3% Black, 7% Asian, 4% Hispanic, 3% other	Exposure and engagement: Eight items assessed ARC social media use related to alcohol marketing (e.g., watched beer, wine, or liquor advertisements on social networking sites, clicked on advertisements for beer, wine, or liquor on social media) from “0 times” to “10 or more times”	Number of days consumed alcohol in past 30 days (+) Number of drinks usually consumed on one occasion (+) Problem drinking index (e.g., frequency of alcohol use causing money problems) with response options from “0 times” to “10 or more times” (+)
Geusens & Beullens, 2021^66^ *Belgium*	128 college students who do not drink heavily	Cross-sectional, survey, researcher-coded posts	65% female, 35% male	Posting: Frequency of sharing photo or video ARC on Instagram in the past 12 months. The scale ranged from 0 = never to 6 = several times a day. Profile evaluation by researchers to identify ARC on Instagram	Past 12-month alcohol use, including frequency of alcohol consumption, ranging from 0 = never to 6 = every day (+); typical number of drinks consumed on a drinking day, ranging from 0 = none to 5 = 10 or more (+); and frequency of binge drinking, ranging from 0 = never to 9 = every day (+)
Burnell et al., 2022^62^ *United States*	232 non-college young adults who do not drink heavily	Cross-sectional, survey, daily diary, researcher-coded posts	61% female, 39% male 60% White, 19% Black, 11% Hispanic, 10% multiracial/other	Posting and exposure: Frequency of exposure to non-ad ARC (baseline survey, 1 = never to 6 = every day; daily survey, 1 = not at all to 4 = in most posts); and frequency of ARC posting (baseline, 1 = never to 6 = every day; daily and past-year posts were coded by researchers)	Frequency of alcohol use in past year (1 = never to 10 = multiple times per day) (+ for baseline and past-year posting, NS for exposure) Daily survey (use alcohol previous day [yes/no]) (NS for posting and exposure)
Cabrera-Nguyen et al., 2016^46^ *United States*	587 non-college young adults who do not drink heavily	Cross-sectional, survey	57% female, 43% male 80% White (non-Latino), 20% other	Posting/engagement: Participant posting pro-alcohol content or following pro-alcohol accounts in the past year, dichotomized to yes/no Exposure: Number of friends who have posted ARC in the past year (options (0) none to (3) almost all then was later dichotomized to yes/no)	Dichotomous engagement in heavy episodic drinking in the past 30 days (+)

* The studies were organized by the outcome measure used, starting with measures used to assess alcohol quantity followed by assessments of alcohol-related consequences.

** Study samples were categorized as drinking heavily if the studies reported mean values of five or more peak drinks/drinks per occasion, heavy episodic drinking, and/or binge drinking. Samples were classified as not drinking heavily if reported mean values were below five peak drinks, and as maybe drinking heavily if not enough information was provided to decide if the study contained a majority heavy-drinking sample or not.

† (+)indicates a statistically significant positive association; (NS) indicates a nonsignificant association. No statistically significant negative associations were found between ARC and drinking outcomes.

*Note*: ARC, alcohol-related content; AUDIT, Alcohol Use Disorder Identification Test;^111^ AUDIT-C, Alcohol Use Disorder Identification Test—Consumption Subscale; BYAACQ, Brief Young Adult Alcohol Consequences Questionnaire; DDQ, Daily Drinking Questionnaire;^113^ FAQ, Facebook Alcohol Questionnaire;^53^ NS, nonsignificant; PDHQ, Personal Drinking Habits Questionnaire;^112^ RAPI, Rutgers Alcohol Problem Index; SM, social media; TLFB, Alcohol Timeline Followback;^97^ TWEAK, Tolerance, Worried, Eye-opener, Amnesia, K-cut down questionnaire;^114^ YAACQ, Young Adult Alcohol Consequences Questionnaire.
